# Early emergence of contractility and mechanotransduction during human megakaryopoiesis

**DOI:** 10.21203/rs.3.rs-10882232/v1

**Published:** 2026-09-29

**Authors:** Oleg V. Kim, Rustem I. Litvinov, Deborah L. French, Lawrence F. Brass, Giulia Pavani, John W. Weisel

**Affiliations:** Virginia Tech; University of Pennsylvania; Children’s Hospital of Philadelphia; University of Pennsylvania; Children’s Hospital of Philadelphia; University of Pennsylvania

**Keywords:** megakaryocyte, megakaryopoiesis, fibrin, clot contraction, mechanotransduction, integrin αIIbβ3

## Abstract

The ability to contract blood clots is a hallmark of functional platelets. Proteins that support agonist-induced platelet contraction are expressed in megakaryocytes (MKs) and transferred to platelets during thrombopoiesis. Here, we investigated when during MK development the functional machinery for contraction emerges. Human induced pluripotent stem cells (iPSCs) were differentiated into embryonic hematopoietic progenitor cells (HPCs) and further matured into MKs (iMKs) to map the emergence of contractility and mechanotransduction across differentiation. Expression of total (CD41) and activated (mAb PAC-1) αIIbβ3 integrin, the thrombin receptor PAR1, and the MK marker CD42b was quantified by flow cytometry. Cell contractility was assessed by optically tracking human plasma clot shrinkage following thrombin stimulation. Undifferentiated iPSCs lacked detectable αIIbβ3 and were non-contractile. Both HPCs and iMKs exhibited agonist-induced contractility, integrin activation, and force transmission to extracellular fibrin, although at different levels. Following thrombin stimulation, activated αIIbβ3 on the cell surface was approximately 5-fold lower in HPCs and restricted to the MK-committed CD42b^+^ subset, indicating that integrin activation is linked to MK commitment. Functionally, both HPCs and iMKs induced clot contraction but with distinct kinetics and magnitude, with HPCs exhibiting approximately 2.5-fold lower contraction rates and final extent. Depletion of CD42b^+^ cells from HPCs reduced contraction to baseline, identifying early MK-lineage progenitors as the principal contractile population at this stage. These findings demonstrate that cellular contractility and αIIbβ3-mediated mechanotransduction emerge early during MK lineage commitment, establishing contractility as an early functional hallmark of megakaryocyte differentiation.

## Introduction

Megakaryocytes (MKs) are polyploid cells that, in the adult, reside in the bone marrow and lungs^1^. Their central role in hemostasis and thrombosis arises from platelet production, as platelets are essential for preventing bleeding following vascular injury and for arterial thrombus formation^2,3^. Beyond platelet production, MKs contribute to a range of (patho)physiologic processes, including inflammation, immunity, bone metastasis and regulation of hematopoietic stem cell (HSC) quiescence^4^.

In addition to other well-known platelet functions, contractility is less understood despite its critical role in hemostasis and thrombosis^5^. In platelets, actomyosin-generated intracellular traction forces transmitted outside through αIIbβ3 integrins (CD41/CD61) compact fibrin networks via cycles of filopodial extension and retraction, stabilizing blood clots and enhancing resistance to deformation and rupture^5–7^. Moreover, platelet-driven contraction consolidates the hemostatic plug and modulates clot permeability, stiffness, and susceptibility to fibrinolysis^8^. Key proteins required for contractile function are synthesized during MK maturation and transferred into nascent platelets during thrombopoiesis^9^. Thus, the protein machinery required for platelet mechanotransduction is established within MKs prior to platelet release, yet inherent contractile function of MKs remains poorly characterized. Contractility of MKs plays a critical role in thrombopoiesis as intracellular traction forces in MKs drive cytoplasmic fragmentation and proplatelet formation^10^. Moreover, our recent work^11^ demonstrates that thrombin-activated iMKs, similar to platelets, induce fibrin clot shrinkage and densification by transmitting contractile forces to the extracellular matrix (ECM). Together, these observations suggest contractility as not only a mechanism for platelet production, but a basic functional property of the MKs linked to hemostasis. This raises an intriguing fundamental question: at what stage during lineage commitment and maturation is this biomechanical functionality acquired?

Addressing this question is challenging because MKs are rare in the bone marrow and exhibit limited *in vitro* differentiation potential and accessibility for functional studies. Human induced pluripotent stem cells (iPSCs) can be efficiently differentiated into embryonic hematopoietic progenitor cells (HPCs), which express αIIbβ3 receptors, and can be further matured into MKs (iMKs)^12,13^. This iPSC-to-HPC-to iMK platform provides a unique and isogenic system to assess contractility across defined stages of MK maturation, enabling studies that are otherwise infeasible with primary human MKs.

Here, we identify the earliest stage of megakaryocyte lineage commitment at which contractility and force transmission emerge. Using a real-time fibrin clot contraction assay, we show that both HPCs and iMKs induce clot shrinkage, with iMKs exhibiting stronger contractility. This distinction correlates with differential expression of the MK maturation marker, GPIbα (CD42b), the thrombin receptor, PAR1, and the active integrin αIIbβ_3_. These findings demonstrate that contractility and mechanotransduction arise prior to full MK maturation and establish a platform for probing their development mechanisms and physiological relevance in hemostasis and thrombosis.

## Results

### Surface markers expression, thrombin responsiveness, and morphological changes during iPSCs-to-HPCs-to-iMKs differentiation

To determine when contractile function emerges during megakaryocyte differentiation, we used an iPSC-based differentiation system in which human induced pluripotent stem cells (iPSCs) recapitulate this early hematopoietic development^14^ and can be differentiated into primitive hematopoietic progenitor cells (HPCs) that can be further matured into a relatively pure iMK population in the presence of stem cell factor (SCF) and thrombopoietin (TPO)^12,13^ (Fig. 1A). To assess contractility development from HPC to mature iMK, two human iPSC lines previously validated for modeling MKs in clot contraction and hematopoietic development^11,13,15,16^ were used. Primitive HPCs were harvested at day 8 and differentiated for an additional 4 days in MK expansion medium to generate iMKs.

Flow cytometry was used to quantify expression of αIIbβ3 integrin (CD41), the megakaryocyte marker CD42b, and the erythroid marker CD235a across iPSCs, HPCs, and iMKs. As expected, CD41, CD42b, and CD235a were undetectable on undifferentiated iPSCs. Primitive HPCs co-expressed CD41 and CD235a, consistent with megakaryocyte-erythroid bipotency, whereas iMKs predominantly exhibited high CD41/CD42b expression with loss of CD235a (Fig. 1B–C). A subset of HPCs also displayed the MK maturation marker CD42b at lower levels compared to iMKs (Fig. 1C), suggesting the presence of an immature iMK-committed population of HPCs. Overall, the average fraction of cells expressing total αIIbβ3 was ~ 70–80% for HPCs and iMKs, with CD41^+^CD42b^+^ fractions averaging ~ 30% in HPCs and ~ 80% in iMKs (Fig. 1D–E).

We next assessed expression of the thrombin receptor PAR1, which contributes to thrombin-induced signaling upstream of αIIbβ3 activation and cell-driven fibrin clot contraction^6,13,17,18^. PAR1 was expressed across all cell populations, but at significantly higher levels in iMKs compared to HPCs (Fig. 2A–C). Within HPCs the MK-committed CD42b^+^ subset exhibited substantially higher (~ 7-fold) PAR1 expression than the CD41^+^CD42b^−^ population (Fig. 2B–C), indicating that PAR1 expression increases during megakaryocyte lineage commitment.

To determine whether αIIbβ3 activation also emerges during this transition, we used the conformation-specific PAC-1 antibody to quantify active αIIbβ3 before and after thrombin stimulation (Fig. 2D)^18^. iPSCs were excluded from this analysis because they lacked detectable αIIbβ3 expression. Following thrombin stimulation, PAC-1 binding was approximately 5-fold lower in HPCs than in iMKs, with PAC-1-positive cells accounting for approximately 10% of HPCs and 50% of iMKs (Fig. 2E). Pre-treatment of the HPCs and iMKs with ethylenediamine tetraacetic acid (EDTA), which dissociates αIIbβ3, prevented PAC-1 binding in both resting and thrombin-stimulated cells. PAC-1 binding was restricted to thrombin-stimulated CD41^+^CD42b^+^ iMKs, as reported previously^17^, and, as shown here, was likewise restricted to the CD41^+^CD42b^+^ subset of HPCs (Fig. 2F). These findings show that thrombin-induced αIIbβ3 activation is linked to megakaryocyte commitment and is already detectable in early CD42b^+^ HPCs, although at lower levels than in mature iMKs.

We next compared the morphology and size of iPSCs, HPCs, and iMKs, using confocal microscopy and scanning electron microscopy, as cell shape reflects the underlying organization of the cytoskeleton. Confocal imaging showed that iMKs were the largest cells, followed by iPSCs and then HPCs, with mean cell diameters of 12.6 ± 1.8 μm, 11.3 ± 2.9 μm, and 9.3 ± 1.9 μm, respectively (Fig. 3A, B). Scanning electron micrographs confirmed broadly similar surface morphology among the cell types and showed characteristic thin filopodial extensions and larger membrane protrusions or blebs, consistent with our previous observations of iMK morphology^11^.

### Contractility emerges in early megakaryocyte-committed progenitors

To assess when functional contractility emerges, fibrin clot shrinkage was monitored over time in PFP containing equal counts of freshly harvested iMKs, HPCs, or iPSCs. Thrombin-stimulated iMKs produced rapid and pronounced clot contraction, while HPCs also induced measurable clot shrinkage but with approximately 2.5-fold lower contraction velocity and final contraction extent compared with iMKs (Fig. 4A–D). iPSCs, used as a negative control, produced only background clot shrinkage of less than 5%.

Because PAC-1 binding and PAR1 expression were enriched in the CD42b^+^ subset of HPCs, we next tested whether this immature megakaryocyte-committed population was responsible for the contractile activity observed in bulk HPCs. Depletion of CD42b^+^ cells from the HPC population caused a marked (~ 4-fold) reduction in clot shrinkage, decreasing contraction close to baseline levels, and increased the contraction lag time by ~ 1.4-fold (Fig. 4E–G). These data identify CD42b^+^ megakaryocyte-committed progenitors as the principal contractile subset within the HPC population.

Together, these results demonstrate that thrombin responsiveness, αIIbβ3 activation, and fibrin-directed contractility emerge before full megakaryocyte maturation. Although mature iMKs display stronger contractility, early CD42b^+^ megakaryocyte-committed HPCs already possess functional mechanotransductive capacity sufficient to drive fibrin clot contraction.

## Discussion

This study identifies an early developmental stage at which megakaryocyte-lineage cells acquire functional contractility and mechanotransduction. The first wave of hematopoiesis occurs in the murine yolk sac and is characterized by the synchronous maturation of lineage-restricted hematopoietic progenitors with megakaryocyte-erythroid potential^19,20^. Using an iPSC-to-HPC-to-iMK differentiation system that models early human hematopoiesis^12,13,14^, we show that undifferentiated iPSCs lack detectable αIIbβ3 and do not contract fibrin clots, whereas both HPCs and iMKs exhibit thrombin-induced αIIbβ3 activation and clot contraction. Importantly, the contractile activity within the HPC population is confined primarily to the CD42b^+^ megakaryocyte-committed subset, indicating that contractility emerges at the onset of megakaryocyte lineage commitment rather than only upon full megakaryocyte maturation.

The acquisition of contractility parallels changes in thrombin responsiveness and integrin activation. Mature iMKs exhibited higher PAR1 expression, greater PAC-1 binding, and stronger clot contraction than HPCs. Within HPCs, PAR1 expression and PAC-1 binding were enriched in the CD42b^+^ subset, linking thrombin responsiveness and αIIbβ3 activation to megakaryocyte commitment. These findings suggest that early megakaryocyte-committed progenitors express a functional PAR1-αIIbβ3 signaling axis capable of transmitting contractile forces to the extracellular fibrin matrix. Nevertheless, compared with mature iMKs, these early progenitors exhibit slower contraction rates and reduced final contraction extent, consistent with lower PAR1 expression and attenuated αIIbβ3 activation.

The absence of substantial contractile activity in CD41^+^CD42b^−^ HPCs suggests that expression of αIIbβ3 alone is not sufficient to confer platelet-like contractile function. Although these cells contain components associated with mechanotransduction, they show limited thrombin-induced integrin activation and do not measurably contract fibrin under the experimental conditions tested. This indicates that additional signaling components, cytoskeletal organization, or lineage-specific maturation events are required for effective force transmission. Although the present data establish strong associations among PAR1 expression, αIIbβ3 activation, CD42b expression, and contractility, further studies will be required to define the causal molecular mechanisms that control acquisition of this biomechanical phenotype. These findings may have implications for early hemostasis during embryonic development and megakaryopoiesis^21^. Specifically, our data raise the possibility that megakaryocyte-committed progenitors may contribute to primitive hemostatic or matrix-remodeling functions before mature platelet production. Beyond development, interactions of MKs with the extracellular matrix may influence hematopoietic niche organization and neovascularization^22^, clot remodeling in inflammatory disorders associated with lung cancer^23^, and non-hematological diseases. Additionally, MK progenitor contractility could be exploited therapeutically, including iMK infusion for thrombocytopenia treatment, and potential applications in regenerative medicine^24,25^.

The results also have practical implications for iPSC-derived megakaryocyte systems. The ability to generate scalable populations of early and mature megakaryocyte-lineage cells provides a platform for identifying molecular programs that regulate the acquisition of contractility. Future proteomic and functional studies comparing CD42b^+^ and CD42b^−^ HPCs with mature iMKs may help define the signaling and cytoskeletal mechanisms that enable platelet-like mechanotransduction. This approach would be difficult to achieve using primary human megakaryocytes, which are rare and less accessible for functional studies.

In conclusion, our study establishes that iMK contractility and αIIbβ3-mediated mechanotrasduction emerge early at the progenitor stage of MK development, prior to full maturation. CD42b + iMK-committed progenitors express PAR1 at levels comparable to mature iMKs, respond to thrombin stimulation, and constitute the principal contractile subset among HPCs. These findings establish contractility as an early functional hallmark of megakaryocyte differentiation and provide a mechanistic and developmental framework for understanding how platelet-like biomechanical cellular function emerges prior to thrombopoiesis.

## Materials and Methods

### Differentiation of iPSCs into HPCs and iMKs

The differentiation of iPSC into primitive HPCs followed by expansion to iMKs was performed as previously described^12^. Briefly, day-8 HPCs were harvested from the supernatant of a differentiation culture and cryopreserved. For iMK generation, HPC were cultured in StemSpan SFEM II (Stemcell Technologies) medium with thrombopoietin (50 ng/mL; R&D) and stem cell factor (25 ng/mL; R&D) for 4 days.

### Flow cytometry and cell sorting of iMKs and their progenitors

Surface expression of CD41 (αIIb subunit of the integrin αIIbβ3), CD42b (vWF receptor GPIbα), CD235a (glycophorin A), and PAR1 were measured by flow cytometry. Antibodies FITC-anti-CD41, PE-anti-CD42b, FITC-anti-CD235a (Biolegend), and AF647-antiPAR1 (R&D) were used. FITC-PAC-1 antibody (BD Biosciences) was used to quantify αIIbβ3 activation, before and after treatment with 1 U/ml human thrombin (MilliporeSigma, T4393) at 37°C for 30 minutes. HPCs were labeled with FITC-anti-CD41 Abs and PE-anti-CD42b Abs and sorted with a BD-FACSJazz using a 100 μm nozzle.

### Blood collection and fractionation

All procedures involving blood from healthy donors were reviewed and approved by the Institutional Review Board (IRB) of the University of Pennsylvania and were conducted in accordance with the approved IRB guidelines. Informed consent was obtained from all participants. Whole venous blood stabilized with 3.2% trisodium citrate (9:1 v/v) was centrifuged at 210g for 15 min at 25°C, within 30 min of collection to obtain platelet-rich plasma (PRP). Platelet-free plasma (PFP) was prepared by centrifugation of PRP at 10,000g, for 15 min at 25°C. Both PRP and PFP samples were used within 3 hours after blood collection.

### Clot contraction assay

iPSCs, HPCs or iMKs were suspended in PFP at 5×10^6^ cells/mL. Plasma clots containing these cells were formed as previously described^11^. Briefly, clot formation and cell activation were induced by adding 2 U/ml human thrombin and 3 mM CaCl_2_ to citrated PFP containing suspended cells. The activated samples were immediately transferred to plastic cuvettes and imaged in light scatter mode using a Thrombodynamics Analyzer (HemaCore). Clot shrinkage was recorded every 30 s for up to 1 hour and images were analyzed computationally to generate contraction kinetics curves, from which average velocity, lag time, and final extent of contraction were extracted.

### Confocal fluorescence microscopy of the cells

iPSCs, HPCs, or iMKs (10^6^ cells in 200 μL each) were fixed with a 4% paraformaldehyde solution (1:1 v/v) and incubation for 20 min followed by 3x washing with PBS, 5-min each. Cells were labeled for F-actin by incubating for 2 hours at room temperature with a Phalloidin-Alexa Fluor 488 (1:1000, Abcam) in PBS containing 1% BSA and 0.1% Triton X-100 for permeabilization. Samples were washed 3x with PBS, mounted in standard mounting medium, and imaged on a Zeiss LSM 880 confocal microscope using a 40× water-immersion objective.

### Scanning electron microscopy (SEM) of the cells

Cell preparations were prepared for SEM as described^11^. Briefly, after incubation in DMEM (ThermoFisher) with or without 1 U/mL thrombin and 3 mM CaCl_2_, isolated cells (10^6^ cells in 200 μL) were fixed with 4% glutaraldehyde (1:1 v/v) in 50 mM cacodylate buffer or PBS for 60 min at room temperature. Fixed samples were settled on polycarbonate filters with 0.1–0.4-μm pore size by centrifugation, washed, dehydrated through graded ethanol, treated with hexamethyldisilazane, and air-dried. Specimens were sputter-coated with Au/Pd and imaged using a FEI Quanta 250 FEG SEM (FEI, Hillsboro).

### Statistical analysis

Statistical analysis was performed in Prism10 (GraphPad) with specific tests described in the figure legends.

## Figures and Tables

**Figure 1:**
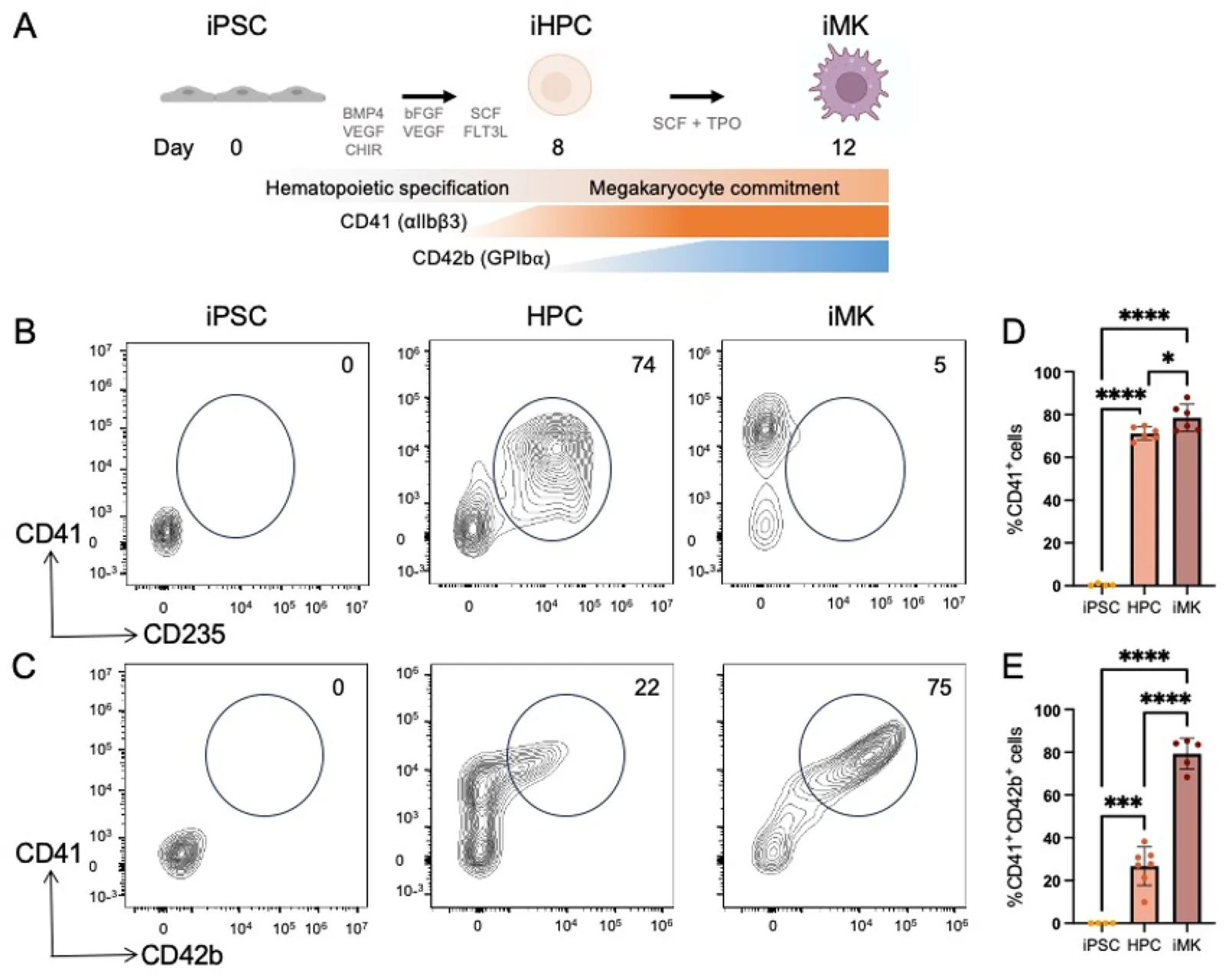
iPSC differentiation to HPC and iMK. (**A**) Schematic representation showing cytokines used and marker expression at different stages of differentiation from iPSCs-to-iHPCs-to-iMKs. (**B-C**) Representative flow cytometry profiles of WT17 iPSCs, HPCs, and iMKs showing co-expression of CD41, CD235a and CD42b. (**D-E**) Quantification of CD41+ and CD41+/CD42b+ populations, respectively (*P<0.05, ***P<10^−3^, ****P<10^−4^, one-way ANOVA with post-hoc Tukey test).

**Figure 2:**
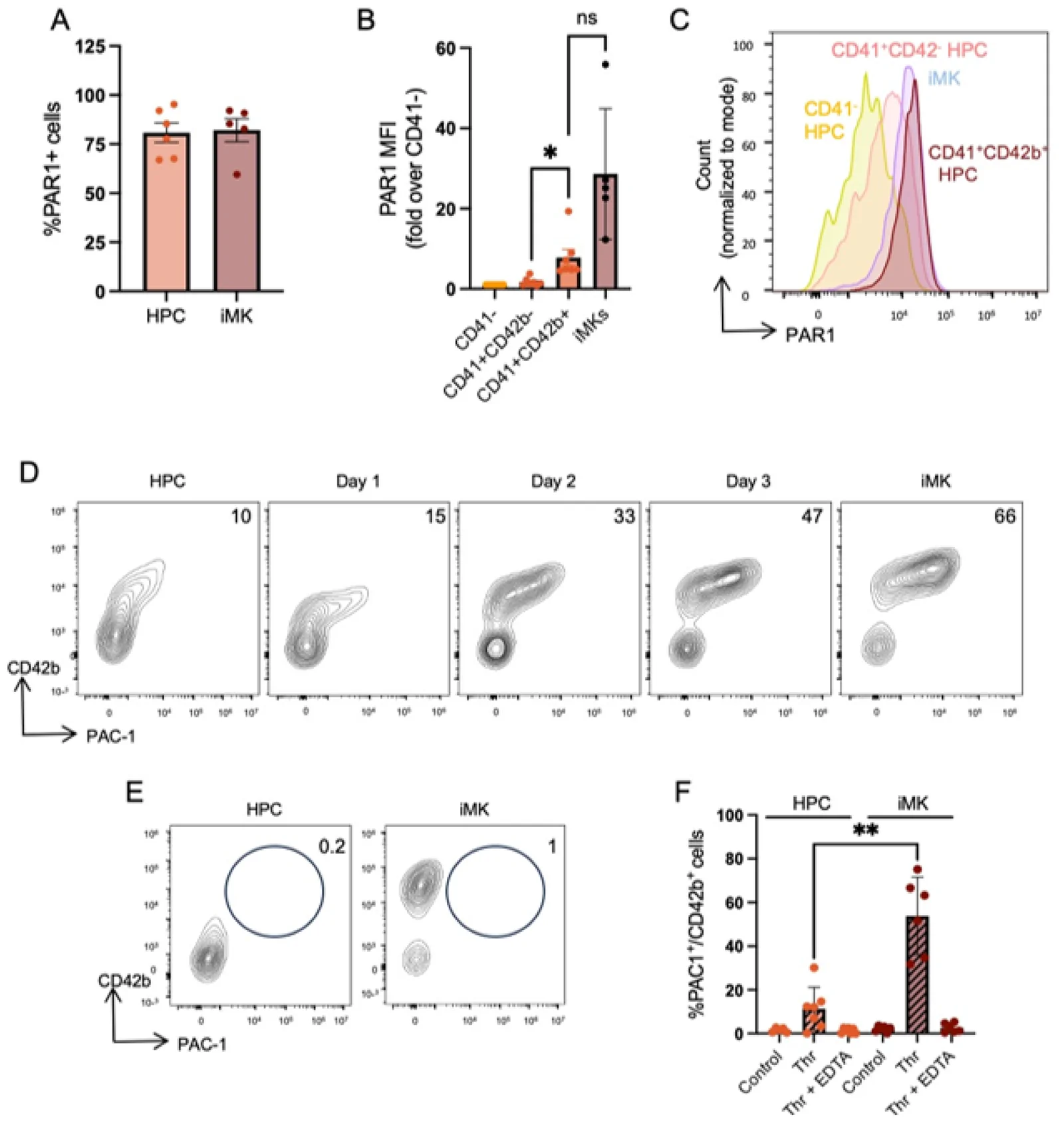
PAR1 expression and PAC-1 binding of WT6 and WT17 HPCs and iMKs. (**A**) Percent PAR1 expression of HPCs and iMKs. (**B**) PAR1 median fluorescence intensity (MFI) of CD41^+^CD42b^−^ and CD41^+^CD42b^+^ HPCs and iMK as fold over CD41^−^ MFI. (**C**) Histogram of PAR1 expression of HPC subsets (negative-yellow, orange-CD41^+^, dark orange-CD41^+^CD42b) and iMKs (light purple). (**D**) Representative flow cytometry profiles of WT17 HPCs and iMKs after thrombin stimulation showing PAC1-binding and CD42b expression over the course of HPC-to-iMK development. (**E**) Representative flow cytometry profiles of unstimulated WT17 HPCs and iMKs. (**F**) Percent PAC-1 binding of unstimulated, thrombin stimulated, and EDTA-pretreated thrombin-stimulated HPCs and iMKs (**P<0.01, two-tailed Mann-Whitney U test).

**Figure 3:**
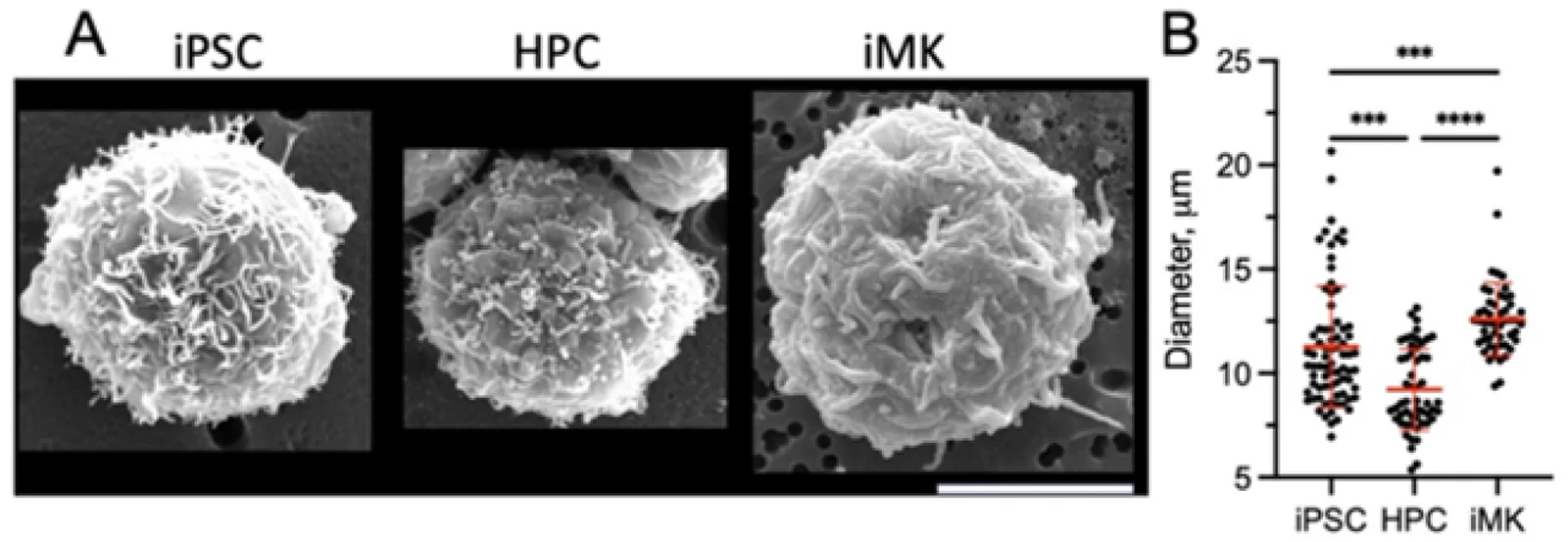
Surface morphology and morphometry of iPSCs, HPCs and iMKs. (**A**) Representative scanning electron micrographs of a WT6 iPSC, HPC, and iMK and (**B**) their size assessment based on confocal microscopy. Statistical significance was assessed using the Kruskal-Wallis with Dunn’s correction for multiple comparison (*P<0.05, **P<10^−2^, ***P<10^−3^, ****P<10^−4^).

**Figure 4:**
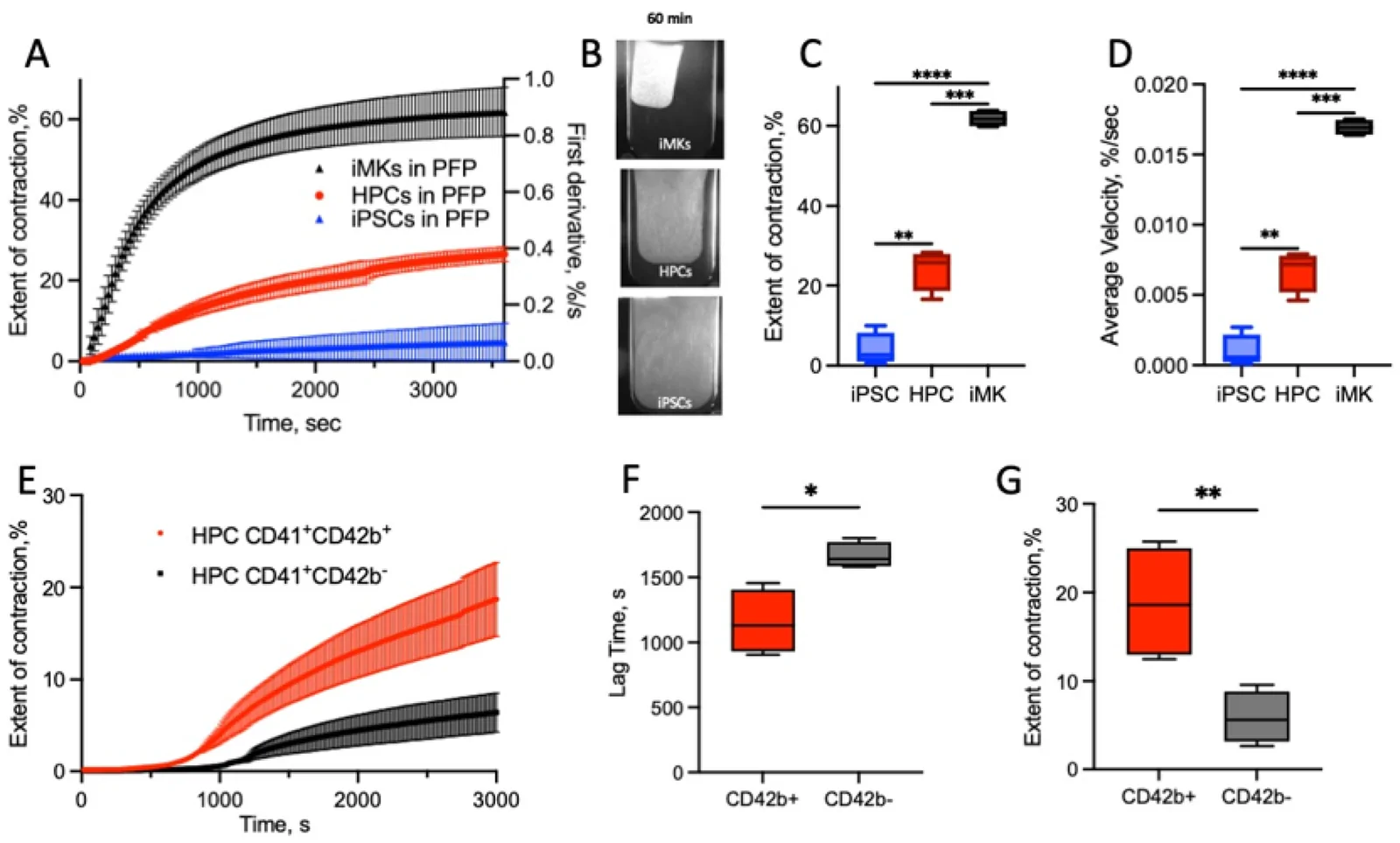
Kinetics of clot contraction showing comparative contractility of WT6 iPSCs, HSCs, and iMKs assessed by the time-dependent shrinkage of plasma clots. (**A**) Averaged contraction kinetic curves of the plasma clots formed in the presence of iPSC, HPC, and mature iMK (M ± SD, n = 4). (**B**) Representative photographic snapshots of platelet-free plasma clots formed and allowed to contract for 60 minutes in the presence of iMKs, HPCs, and iPSCs. (**C, D**) Plots showing the final 60-min-extent of contraction and average contraction velocity for the clots formed in the presence of iPSC, HPC, and mature iMK. Results are presented as median and IQR. Statistical significance assessed using the Welch-ANOVA test (*P<0.05, **P<10^−2^, ***P<10^−3^, ****P<10^−4^). (**E**) Averaged contraction kinetic curves for the clots formed in the presence of HPCs with or without CD42b+-cell depletion (M ± SD, n = 4). (**F, G**) Lag time and final extent of contraction corresponding to (E) (median with IQR). Statistical significance was assessed using the Mann-Whitney U test (*P<0.05, **P<10^−2^).

## Data Availability

All data supporting the findings of this study are available within the article or from the corresponding authors upon reasonable request.
